# Recent technology development in radiotherapy for intracranial meningiomas

**DOI:** 10.3389/fneur.2025.1568898

**Published:** 2025-04-28

**Authors:** Rebecca Ungar, Joshua Kilian-Meneghin, Brett Eckroate, Sabin Motwani, Ning Yue, Ke Nie, Zhenyu Xiong, Yin Zhang

**Affiliations:** ^1^Department or Physics and Astronomy, Rutgers University, Piscataway, NJ, United States; ^2^Department of Radiation Oncology, Rutgers Cancer Institute, New Brunswick, NJ, United States

**Keywords:** radiotherapy, brachytherapy, proton therapy, GammaKnife, CyberKnife, artificial intelligence, meningiomas

## Abstract

Meningiomas are the most common central nervous system (CNS) tumors in the United States. Radiotherapy plays an important role in the management of meningiomas and has been established as an effective means of local tumor control. The recent technology development in artificial intelligence, understanding of meningioma biology and molecular imaging, will likely impact the clinical management of meningiomas, including treatment efficacy, efficiency and safety. This review summarizes recent technological advances that may influence radiotherapy management for meningiomas, including external beam radiation therapy, proton therapy and brachytherapy.

## Introduction

Meningiomas are the most common central nervous system (CNS) tumors, representing over 35% of primary CNS tumors in the United States (1). They originate from arachnoid cells, which form the middle protective layer of the brain, and typically grow slowly. Since 1993, the World Health Organization (WHO) has classified meningiomas into three histological grades: WHO Grade I (benign), WHO Grade II (atypical), and WHO Grade III (malignant). The WHO grading system was revised in 2000, 2007, 2016, and 2021, with Grade I tumors accounting for ~80% of cases, Grade II for ~18%, and Grade III for ~2% when following 2021 revision (2).

Clinical management of Meningioma depends on factors such as patient age, tumor size, histological grade, and molecular data. Surgery remains the primary treatment for symptomatic Grade I meningiomas amenable to resection, whereas small asymptomatic Grade I lesions can often be observed (3). Complete surgical resection remains the goal; however, about one-third of meningiomas are unresectable due to tumor location or other considerations (4). Radiotherapy (RT) is instrumental, particularly when resection is impossible or incomplete, high-grade disease is present, or tumors recur. Extensive evidence supports RT for Grade I disease (5–8), and current NCCN guidelines recommend RT as a primary treatment in selected Grade I meningiomas and postoperatively for incompletely resected Grade II and all Grade III tumors (9). A range of RT techniques—including three-dimensional conformal radiotherapy (3D-CRT), intensity-modulated radiotherapy (IMRT), stereotactic radiosurgery (SRS), fractionated stereotactic radiotherapy (SRT), brachytherapy and proton therapy—are used in meningioma treatment. This review summarizes recent technological advances that may influence radiotherapy management for meningiomas.

## External beam radiotherapy

Conventional LINAC-based radiotherapy has become increasingly important for meningioma management, particularly when complete resection is not feasible. High-energy photon beams produced by a linear accelerator can be precisely shaped and directed using advanced image guidance and multi-leaf collimators. The LINAC’s isocentric setup aligns the gantry, couch, and collimator around a single point, facilitating non-coplanar beam angles that minimize radiation exposure to surrounding normal tissues. Employing flattening filter-free (FFF) beams can further increase dose rates, thereby shortening treatment durations.

SRS and SRT using conventional LINAC systems offer versatility for a wide spectrum of tumor sizes and locations. A notable development is Varian HyperArc, an automated non-coplanar planning and delivery system. By combining multiple optimized arcs with automated planning algorithms, HyperArc refines dose conformity for complex or multiple lesions while limiting normal tissue exposure. In a dosimetric study by Snyder et al. (10), 12 patients with skull base meningiomas were retrospectively planned using HyperArc. The resulting treatment plans achieved high target coverage with decreased normal brain dose and superior treatment efficiency.

Numerous retrospective analyses confirm the efficacy and safety of LINAC-based SRS for WHO Grade I meningiomas. Pou et al. (11) found 100, 98.4, and 92.6% local control at 1, 5, and 10 years, respectively, in 60 patients. Another study of 241 patients with skull base meningiomas revealed a 91.2% tumor control rate over a median follow-up of 102 months, with 5-, 10-, and 14-year progression-free survival (PFS) exceeding 85% (12). Gawish et al. (13), in a cohort of 36 patients, observed 95% local control at 2 years and 70% at 5 years. Alatriste-Martínez et al. (14) reported a 93% local control rate over 68 months, confirming that LINAC-based SRS or SRT can yield durable outcomes with minimal morbidity.

LINAC-based therapy also benefits atypical meningiomas and those in surgically challenging locations. Ortiz García et al. (15) demonstrated 95% local tumor control in cerebellopontine angle meningiomas, with an average volume reduction of 32.8% over nearly 87 months. A European Phase II study [EORTC 22042-26042 (16)] is examining high-dose postoperative radiotherapy for atypical and malignant meningiomas; preliminary findings suggest that dose escalation can improve tumor control with manageable toxicity. Zeng et al. (17) retrospectively evaluated 118 patients with WHO Grade II or III disease, comparing standard-dose (<66 Gy) and escalated-dose (≥66 Gy) radiotherapy. The escalated-dose group showed higher progression-free survival rates—78.9, 72.2, and 64.6% at 3, 4, and 5 years, respectively—with reduced local failure.

**Figure 1:**
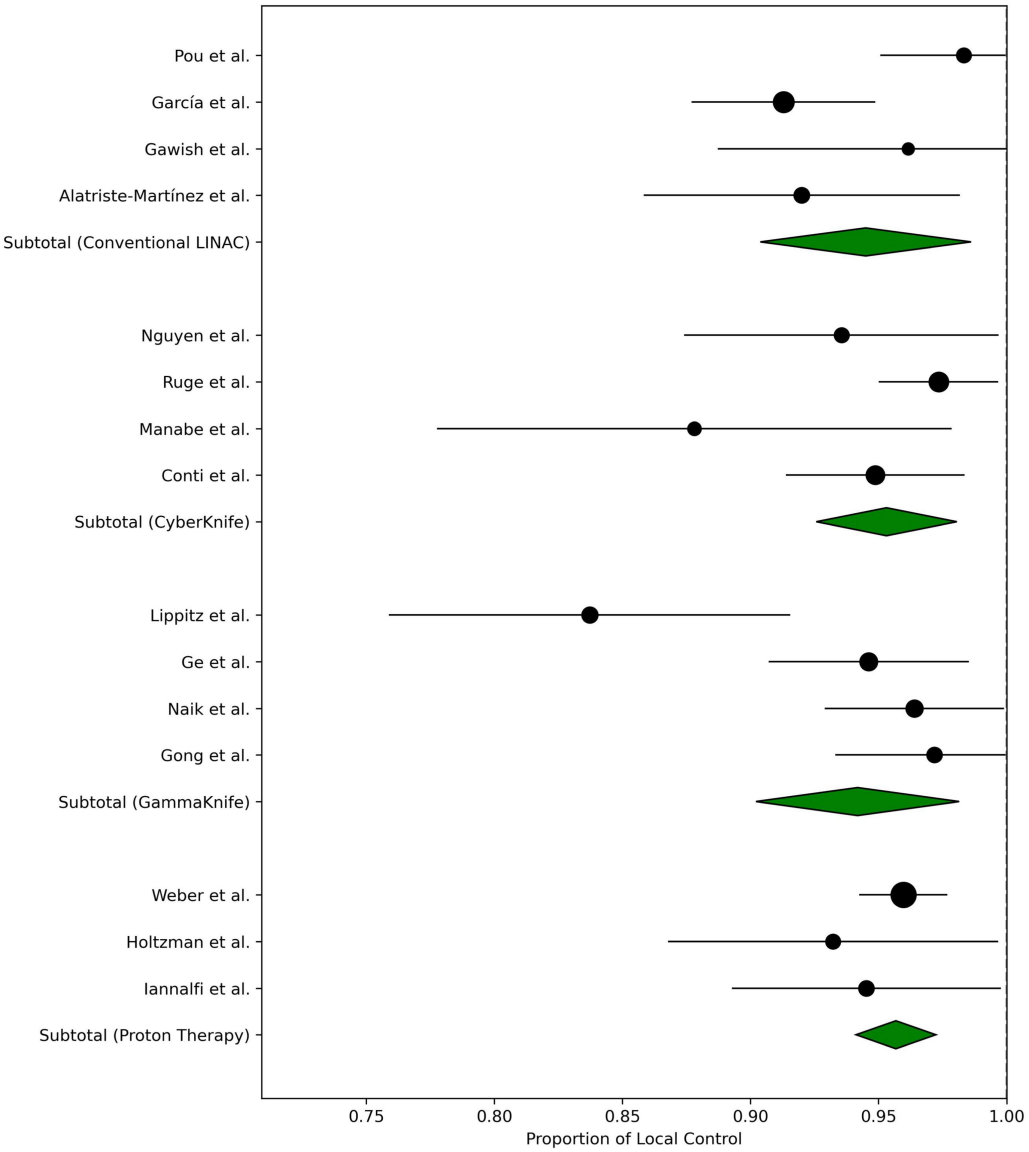
Forest plot of single-arm local control rates for WHO Grade I meningiomas treated with various radiation modalities (conventional LINAC, CyberKnife, GammaKnife, and proton therapy). Brachytherapy was not included here because the majority of brachytherapy studies focus on recurrent or high-grade meningiomas. Each circle represents an individual study’s point estimate, with the horizontal bar indicating its 95% confidence interval, and the size of the circle proportional to the study’s sample size. The green diamond shows the pooled estimate (with a 95% confidence interval) for each modality, derived using a random-effects meta-analysis. A vertical dashed line at x = 1.0 indicates a 100% local control reference.

Several prospective trials further investigate the role of postoperative radiotherapy in meningioma management. RTOG 0539 [ClinicalTrials.gov Identifier: NCT00895622 (18)] is a phase II, risk-stratified study assessing fractionated external beam radiotherapy for diverse meningioma subgroups after resection. NRG-BN-003 (ClinicalTrials.gov Identifier: NCT03180268), a phase III trial, examines whether adjuvant radiotherapy can improve outcomes in newly diagnosed atypical meningiomas. Results from these and other investigations will likely refine standard-of-care guidelines and optimize local tumor control strategies across different meningioma grades. In Figure 1, the recent published data on local control rates of Grade I meningiomas using different modalities is summarized.

CyberKnife (CK) is a specialized robotic stereotactic treatment system designed to deliver high-precision, image-guided radiation treatment. It features a compact LINAC mounted on a multi-axis robotic arm, enabling radiation delivery from numerous non-coplanar angles. Unlike conventional C-Arm LINAC systems, CK near-continuously tracks the tumor using orthogonal x-ray imaging, thereby detecting and correcting any motion during treatment. This near real-time tracking, combined with submillimeter accuracy, allows clinicians to deliver prescription doses while sparing nearby critical structures such as the optic apparatus, cranial nerves, and sensitive brain regions. CK also supports hypofractionated regimens, making it suitable for tumors considered too large or complex for traditional radiosurgery. Numerous clinical studies report favorable local control, and low toxicity, supporting CK’s role as a minimally invasive alternative or complement to surgery.

Several retrospective investigations highlight CK’s effectiveness for small, benign WHO Grade I meningiomas. Nguyen et al. (19) evaluated 62 patients (67 meningiomas) treated with SRT, finding 94.4% local control over a median follow-up of 64.7 months, with 5-year PFS and overall survival (OS) of 85.2 and 91.0%, respectively. Ruge et al. (20) reported 97.7% local control among 188 patients (218 meningiomas) treated with single-fraction SRS. Manabe et al. (21) studied 41 patients with WHO Grade I intracranial meningiomas treated with CK-based SRS or SRT, achieving local tumor control in 86% of cases over a median follow-up of 49 months. Another investigation (22) involving 156 skull base meningiomas treated with multisession SRS attained 95% local tumor control at 36.6 months, with 2-, 5-, and 10-year PFS rates of 95, 90, and 80.8%. Collectively, these findings confirm robust tumor control and minimal morbidity in Grade I disease.

CK also shows promise for complex, inoperable meningiomas. In 13 patients with olfactory groove meningiomas (OGMs), Liu et al. (23) found 100% local control over 48 months, with a median tumor volume reduction of 31.7%. Among 167 patients with perioptic meningiomas (24), local control reached 95.2% at a median follow-up of 51 months, along with high PFS rates at 3, 5, and 8 years. Malone et al. (25) reported on a single case of an unresectable foramen magnum meningioma treated with 30 Gy in 5 fractions, achieving 8-year local control and a 30% reduction in tumor volume without adverse effects. Hong et al. (26), in a study of 113 patients with central skull base meningiomas, observed 98% local control at 49 months, highlighting CK’s capacity to deliver therapeutic doses while preserving neurological function.

Although high-grade meningiomas are more challenging, CK can still provide meaningful tumor control with acceptable toxicity. Acker et al. (27) demonstrated local control rates of 97, 77, and 67% at 12, 36, and 60 months, respectively, for WHO Grade II lesions, and 66% at 12 and 24 months for Grade III lesions. In another study (28) of 102 WHO Grade II and 4 WHO Grade III meningiomas, 45% of lesions remained stable over time, with mild toxicities and no reported radionecrosis. These data underscore CK’s viability for a wide range of meningioma grades, particularly when surgical resection is not possible or is incomplete.

GammaKnife (GK) is a dedicated stereotactic radiosurgery platform that uses multiple cobalt-60 sources to deliver highly focused *γ*-ray beams for intracranial lesions, including meningiomas. Advances in imaging, planning software, and delivery techniques facilitate both single-fraction and hypofractionated treatment protocols. The introduction of newer GK platforms, such as GammaKnife Icon, has further broadened therapeutic capabilities through on-board imaging and frameless immobilization, enabling fractionated stereotactic treatment on this platform.

A lot of data supports GK’s efficacy for benign WHO Grade I meningiomas. In a 10-year retrospective study, Lippitz et al. (29) reported 87.8% local tumor control among 86 patients (130 tumors). Ge et al. (30), analyzing 130 patients treated with GK SRS, noted a 94.6% tumor control rate over a median follow-up of 36.5 months, with neurological symptoms improving in almost one-third of patients. Naik et al. (31) documented 96.3% control in Grade I lesions, dropping to 62.5% in Grade II, illustrating the impact of histology on outcomes. A dose-staged approach (32) achieved 97.2% local control over 36 months, with no significant long-term neurological deficits. Collectively, these studies validate GK as a safe and effective modality for benign meningiomas, especially when resection is incomplete or poses excessive risks.

GK has also been shown effective for meningiomas in rare or difficult locations. Daza-Ovalle et al. (33) reported a 94.7% local control rate for intraventricular meningiomas, with 5- and 10-year PFS of 95 and 85%. Hung et al. (34) recorded 92% control for cavernous sinus meningiomas, while Abdallah et al. (35) documented 100 and 83% local control at 5 and 10 years for lesions at the confluence of the falx and tentorium. Akyoldas et al. (36) attained 100% control for anterior clinoid process meningiomas over 75 months, and Ha et al. (37) reported a 91.1% PFS at 5 years for petroclival meningiomas, dropping to nearly 70% at 10 years. These outcomes highlight GK’s capacity to spare vital structures while delivering definitive treatment in anatomically challenging scenarios.

For higher-grade tumors (WHO Grade II and III), GK SRS can serve as an adjunct or alternative when complete resection is unfeasible. Migliorati et al. (38) evaluated 47 WHO Grade II meningiomas and found that among 24 treated with GK for residual or recurrent lesions, 87.5% remained controlled at 20–23 months. Kim et al. (39) reported a 50% local control rate over a median follow-up of 106.5 months in recurrent or residual Grade II and III lesions, illustrating the difficulty posed by higher-grade disease. Although these rates are lower compared to those for benign disease, GK SRS can still offer meaningful tumor stabilization and palliation when surgery is limited by anatomical constraints or patient factors.

## Particle therapy

Proton therapy (PT) is an advanced form of radiotherapy that employs protons to treat tumors and other diseases. In contrast to photon- or electron-based radiotherapy, proton beams exhibit distinct physical properties, notably a finite range and steep dose gradient at the Bragg peak. As protons travel through tissue, their energy deposition increases at a specific depth (the Bragg peak), minimizing exit dose and thereby limiting irradiation of normal tissues. This characteristic can be especially valuable for meningiomas located near critical structures such as the brainstem, optic apparatus, and hippocampus.

A comparative planning study (40) analyzing intensity-modulated proton therapy (IMPT), volumetric arc therapy (VMAT), and IMRT for skull-base meningiomas found that IMPT significantly improved dose conformity and reduced bilateral hippocampal and mean brain dose. In a review by Weber et al. (41), about 500 WHO Grade I meningioma patients treated with upfront PT or salvage PT demonstrated excellent local control and minimal toxicities, although Grade II and III tumors required more careful evaluation. Holtzman et al. (42) reported outcomes in 59 WHO Grade I patients who received a median dose of 50.4 Gy RBE (relative biological effectiveness), showing an 87% 5-year actuarial OS and a 2% rate of Grade ≥3 toxicities. Iannalfi et al. (43) retrospectively examined 167 meningioma patients receiving pencil-beam scanning (PBS) PT. Among these, 73 were WHO Grade I, 26 were Grade II, and 3 were Grade III. The median prescribed dose was 55.8 Gy for benign/radiologically diagnosed meningiomas and 66 Gy for atypical/anaplastic lesions. Five-year local recurrence-free survival (LRFS) was 98% for benign/radiologically diagnosed tumors and 47% for higher-grade lesions, with limited Grade 3 toxicities and no Grade 4 or 5 events.

Proton therapy also has applications in re-irradiation settings. Imber et al. (44) studied 16 patients (7 WHO Grade I, 8 Grade II, and 1 Grade III) who underwent PT re-irradiation. The median dose was 60 Gy RBE, yielding 1-year and 2-year PFS rates of 80 and 43%, respectively. In a larger series by Scartoni et al. (45) 32 recurrent meningioma patients re-irradiated with PBS reported similar outcomes using median doses around 54 Gy RBE.

With the emergence of advanced imaging, prognostic biomarkers may improve patient stratification. Buizza et al. identified diffusion-weighted MRI parameters—such as diffusion coefficient and apparent cellularity—that can aid in assessing tumor grading and therapy response at a microscale for patients who receive PT (46, 47). However, PT remains costly, and more robust evidence is awaited from ongoing trials (ClinicalTrials.gov Identifier: NCT01117844, NCT02693990, and NCT02978677) that are evaluating its efficacy for meningioma management.

## Brachytherapy

Brachytherapy (BT) involves placing radioactive sources in or near the target tissue. In the context of recurrent high-grade meningiomas, permanent seed implants with isotopes like Iodine-125 (I-125) or Cesium-131 (Cs-131) have been used as adjuvant therapy after resection. Cs-131, a newer isotope with a shorter half-life (9.7 days vs. 59.4 days for I-125) and lower mean energy of 30 keV, rapidly delivers half its therapeutic dose within the first 10 days. Both isotopes emit low-energy photons, limiting tissue penetration to a few millimeters, making them suitable only when minimal residual tumor remains. BT offers several theoretical benefits for recurrent meningioma: (1) reduced radiation exposure to normal tissue by using low-energy photon-emitting isotopes; (2) immediate radiotherapy initiation post-resection, when tumor burden is lowest; and (3) intraoperative placement of sources for precise targeting. Multidisciplinary collaboration among neurosurgery, radiation oncology, and medical physics is crucial for planning and performing BT implants.

Magill et al. documented outcomes for 42 patients (50 resections) with recurrent high-grade meningiomas treated with I-125 BT (48). The median time to progression after resection plus I-125 BT was 20.9 months for atypical and 11.4 months for malignant meningiomas. Median survival was 3.5 years and 2.3 years, respectively, and 19% of patients experienced radiation necrosis. In a separate series using Cs-131 seeds, Chen et al. treated 12 recurrent atypical (*n* = 7) or anaplastic (*n* = 5) meningiomas with a prescription dose of 80 Gy to 5 mm from the resection cavity using stranded Cs-131 seeds (49). One-year local control was 100%, and OS reached 91.7%. Bander et al. similarly used Cs-131 with a prescription dose of 80 Gy for 15 patients (Grade I–III), noting 73.3% 1-year survival and 83.3% local control (50). Another single-center retrospective study by Mooney et al. employed both I-125 and Cs-131 seeds embedded in an absorbable mesh to achieve a 100 Gy minimum peripheral dose at 5 mm from the cavity margin for 11 recurrent high-grade meningiomas (51). Six-month and 12-month PFS rates were 92.3 and 84.6%, respectively. These patients demonstrated improved PFS compared with controls without BT, although at the cost of a higher incidence of radiation necrosis.

GammaTile, as an emerging BT platform, using a modular resorbable collagen-based seed carrier with radioisotope Cs-131, has attracted interest to treat recurrent meningioma, glioblastoma, and brain metastasis (52). The dimension of each tile is 2 cm x 2 cm x 4 mm with four Cs-131 titanium-encased sources (52). The center of each Cs-131 seed is spaced 1 cm apart from one another and the seeds are spaced 3 mm from the surface of the tile that faces the brain parenchyma. Unlike traditional loose seeds or stranded seeds, the GammaTile consists of a layer of collagen, which serves as a spacer to avoid direct contact of Cs-131 seeds with the brain parenchyma, potentially reducing the risk of high dose induced necrosis. The design and the adherence of the collagen matrix can minimize seed migration after implantation and maintain the source spacing after placement. Brachman and colleagues studied the combination of maximum safe resection and adjuvant RT using permanent intracranial BT GammaTile in patients with recurrent, previously irradiated aggressive meningiomas, with median BT radiation dose of 63 Gy (range 54–80 Gy) (53). This study included 19 patients with 20 recurrent tumors, including 4 WHO grade I, 14 WHO grade II, and 2 WHO grade III. At a median radiographic follow-up of 15.4 months (range 0.03–47.5 months), local failure occurred in 2 cases within 1.5 cm of the implant bed. This study demonstrated surgery and BT with GammaTile to be a safe and effective treatment option for recurrent aggressive meningiomas. There is an ongoing registry study (ClinicalTrials.gov Identifier: NCT04427384) to evaluate real-world clinical outcomes and patient reported outcomes that measure the effectiveness and safety of using GammaTiles on brain tumors, including meningiomas.

Each modality for treating meningiomas demonstrated its own distinct advantages and limitations. For example, even though proton therapy shows beneficial dosimetry in treatment planning, this treatment modality is not easily accessible as other photon-based treatment method and the cost for proton therapy would be higher. For brachytherapy, the accessibility for meningioma management is also inferior to other modalities and it is an invasive procedure. Due to the unique geometry in machine, the GK beam showed sharper penumbra, resulting in quicker dose fall-off in plan dosimetry to spare health brain tissue. CK would allow quicker treatment, comparing with GK, due to higher dose rate in general (Table 1).

**Table 1:** The accessibility of different radiation therapy (RT) modalities for meningioma management.

Treatment modality	Patient cohort	Accessibility
Conventional LINAC	Grade I – III	Normal
CK	Grade I – III	Moderate
GK	Grade I – III	Moderate
Proton therapy	Grade I – III	Limited
Brachytherapy	Recurrent	Limited

## Emerging technologies

In the past few years, we have witnessed rapid advancements of artificial intelligence (AI) in the field of medicine, including radiation oncology. Machine learning (ML) and deep learning (DL) models, such as convolutional neural network (CNN), have been built and evaluated as assisting tools in the workflow of RT, including image registration, image segmentation, automatic planning, dose distribution prediction, and outcome prediction (54). AI also has the potential to extract quantitative imaging features from regular clinical images, including CT, MRI, PET and other advanced imaging techniques. These features, which quantify image characteristics, can be combined with clinical parameters or molecular markers to create mathematical or machine learning models. This process of developing models based on quantitative features derived from medical images is known as radiomics, which allows the connection of the imaging phenotype of the tumor to the molecular and biological characteristics (55). A few studies on radiomics have concentrated on the application of ML/DL approaches for the preoperative prediction of meningioma grading using MRI images. Utilizing radiologic and radiomic features, including apparent diffusion coefficient and sphericity, extracted from preoperative MR images, Morin et al. developed prognostic models for tumor grade, local failure (LF), and OS in meningioma patients (56). Compared to models based on clinical features alone, models combining clinical, radiologic and radiomic features were demonstrated to have improved prediction accuracy for meningioma grade, LF, and OS. The same group of researchers developed integrated models taking into consideration of all available demographic, clinical, radiographic and pathologic data (57). With the integrated models, decision trees and nomograms were developed to identify patients with different risks of LF or OS. The predictive models can be used as a decision support tool to provide individualized patient care.

Functional imaging modalities, including positron emission tomography (PET), are of particular interest for the detection and spatial assessment of meningiomas. In the vast majority of meningiomas of all grades, the overexpression of the somatostatin receptors (SSTR) is present (58, 59). A couple of radiolabeled PET tracers targeting SSTR have been evaluated for assisting accurate target delineation, including ^68^Ga-DOTA-Tyr3-octreotide (^68^Ga-DOTATOC) (60, 61), ^68^Ga-DOTA-D-Phe1-Tyr3-octreotate (^68^Ga-DOTATATE) (62), and ^68^Ga-DOTA-l-Nal3-octreotide (^68^Ga-DOTANOC) (63). Multiple studies supported the use of PET imaging with SSTR tracers for meningioma target volume delineation in treatment planning, especially for SRS/SRT treatment where the margin is smaller. A joint practice guideline by EANM/EANO/RANO/SNMMI for diagnostics and therapy of meningiomas using radiolabeled SSTR ligands was recently published to harmonize data acquisition and interpretation across centers and facilitate comparability studies (64). A recent consensus report on target volume delineation guidelines for meningiomas receiving postoperative radiation using ^68^Ga-DOTATATE PET/CT images was published (65). Targeting the overexpressed SSTR, radiopharmaceuticals with ^67^Cu-labeled, ^90^Y-labeled and ^177^Lu-labeled SSRT ligands have been actively studied as a salvage treatment option for meningioma (66–68). Other novel molecular imaging and theranostic tracers are being developed and evaluated to better understand meningioma biology to assist clinical decision-making.

## Conclusion

RT plays a pivotal role in the clinical management of meningiomas. The recent technology development, especially in AI, understanding of meningioma biology and molecular imaging, will likely impact the clinical management of meningiomas, including treatment efficacy, efficiency and safety. The clinical trials will help to guide the use of radiation therapy in newly diagnosed and recurring meningiomas.
